# Functional Genome Annotation by Combined Analysis across Microarray Studies of *Trypanosoma brucei*

**DOI:** 10.1371/journal.pntd.0000810

**Published:** 2010-08-31

**Authors:** Hamed Shateri Najafabadi, Reza Salavati

**Affiliations:** 1 Institute of Parasitology, McGill University, Ste. Anne de Bellevue, Quebec, Canada; 2 McGill Centre for Bioinformatics, McGill University, Montreal, Quebec, Canada; 3 Department of Biochemistry, McGill University, Montreal, Quebec, Canada; University of Pittsburgh, United States of America

## Abstract

**Background:**

Functional annotation of trypanosomatid genomes has been a daunting task due to the low similarity of their genes with annotated genes of other organisms. Three recent studies have provided gene expression profiles in several different conditions and life stages for one of the main disease-causing trypanosomatids, *Trypanosoma brucei*. These data can be used to study the gene functions and regulatory mechanisms in this organism.

**Methodology/Principal Findings:**

Combining the data from three different microarray studies of *T. brucei*, we show that functional linkages among *T. brucei* genes can be identified based on gene coexpression, leading to a powerful approach for gene function prediction. These predictions can be further improved by considering the expression profiles of orthologous genes from other trypanosomatids. Furthermore, gene expression profiles can be used to discover potential regulatory elements within 3′ untranslated regions.

**Conclusions/Significance:**

These results suggest that although trypanosomatids do not regulate genes at transcription level, trypanosomatid genes with related functions are coregulated post-transcriptionally via modulation of mRNA stability, implying the presence of complex regulatory networks in these organisms. Our analysis highlights the demand for a thorough transcript profiling of *T. brucei* genome in parallel with other trypanosomatid genomes, which can provide a powerful means to improve their functional annotation.

## Author Summary

The impact of the trypanosomatid genome sequencing projects, especially *Trypanosoma brucei*, *Trypanosoma cruzi*, and *Leishmania* species, has been substantial. However, significant numbers of hypothetical or conserved hypothetical trypanosomatid-specific genes remain uncharacterized with possible roles in infectivity and disease. The genes in trypanosomatids are organized into large multigenic clusters that are transcribed as polycistronic transcription units, thus limiting gene regulation mainly to the post-transcriptional level. In this study, we examined three independent DNA microarray studies of *T. brucei*, and found that mRNA abundances of functionally related *T. brucei* genes are co-regulated, most probably via post-transcriptional events. We then used this property of functionally related genes to predict the functions of hypothetical genes in *T. brucei*. The predictions were further improved by including DNA microarray studies from the closely related trypanosomatid *Leishmania infantum*, suggesting that parallel transcript profiling of trypanosomatids will be of considerable potential for refining gene function predictions in these organisms. Furthermore, we found that potential regulatory elements within untranslated regions of RNA transcripts can be predicted based on combined expression data from different microarray studies. Overall, this approach contributes to a better understanding of the mechanisms underlying gene regulation and function in trypanosomatids.

## Introduction

*Trypanosoma brucei*, the causative agent of human sleeping sickness, is one of the major disease-causing trypanosomatids whose genome sequences have been determined for about five years [1]. However, the functions of most of the genes of this parasite still remain unknown, mainly because of the poor similarity between their sequences and the sequences of characterized genes from other organisms. This highlights the need for employing homology-independent approaches to improve the functional annotation of *T. brucei* genome. Since co-expressed genes tend to share similar functions, belong to the same pathways, or participate in the same processes [2], the function of a gene can often be predicted based on the functions of the genes it is co-expressed with [3]. This provides a powerful homology independent method for functional annotation of a genome.

In *T. brucei*, most genes are not transcriptionally regulated [4], [5]. Instead, genes are transcribed as polycistronic mRNAs [6] that heavily depend on post-transcriptional processes for maturation and regulation. Some reports suggest that this lack of transcriptional regulation results in limited responsiveness of *T. brucei* transcriptome to altered environment and genetic background [7], thus, preventing the construction of an informative coexpression network. Nevertheless, recent studies have reported that mRNAs of *T. brucei* genes with related functions share similar sequence motifs in their untranslated regions (UTRs), suggesting that they are coregulated at post-transcriptional level via common sequence-dependent mechanisms for regulation of mRNA stability and/or translation [8].

Three recent studies have provided genome-wide expression profiles for procyclic form (PF) and bloodstream form (BF) *T. brucei* during differentiation [9]–[11]. Here, we demonstrate that while the data from each of these individual studies is not significantly informative about gene function, their collection can be used to construct a coexpression network that reflects the functional linkages among genes. We have used this coexpression network to predict the broad functions of several currently uncharacterized *T. brucei* genes, and have expanded our predictions by considering coexpression relationships that are conserved between *T. brucei* and *Leishmania infantum*. Finally, we show that by combining the expression data from the microarray studies of *T. brucei*, we can cluster the genes based on expression profiles and use these clusters to identify potential regulatory elements within mRNA untranslated regions.

## Methods

The methods that we have used in this study are summarized in this section. The details of the methods are provided in Supplementary Methods S1.

### Data sources

We used *T. brucei* mRNA expression data from three recent publications [9]–[11]. A set of 7488 *T. brucei* genes was shared by these three studies, each gene represented by a total of 17 expression values: four from ref. [9], eight from ref. [10] and five from ref. [11]. The functional annotations of *T. brucei* genes were obtained from KEGG pathway database [12] and TriTrypDB [13]. The sequences of 3′ UTRs were extracted based on previous splice-site predictions [14]; sequences were either used completely or truncated to contain only the first 1000nt in the 5′ end of the 3′ UTR. For identification of conserved coexpression, we used a collection of *Leishmania infantum* gene expression profiles from three different studies [15]–[17]. Orthologous genes between *T. brucei* and *L. infantum* were identified based on their protein sequences, obtained from KEGG [12].

### Construction and evaluation of a coexpression network based on *T. brucei* microarray studies

The coexpression values for ∼2.8×10^7^ *T. brucei* gene pairs, measured as Pearson correlation coefficients across several experiments, were obtained using different experiment sets: (i) a set of four experiments from ref. [9], (ii) a set of eight experiments from ref. [10], (iii) a set of five experiments from ref. [11], (iv) the set of all the 17 experiments from these three studies, and (v) a selected subset from the 17 experiments; this subset was chosen so as to maximize the accuracy and coverage of predicting functional linkages, as explained in the next section. Gene pairs with correlation coefficients greater than a specified threshold were used to construct the coexpression networks. This threshold was chosen so that at least 75% of linkages in the coexpression network would represent functional linkages according to KEGG (in other words, the coexpression network would have a precision of 75%).

### Selecting an optimum subset of microarray experiments for identification of functional linkages

Different microarray studies may present data that do not equally correlate with functional linkages; inclusion of experiments that do not reflect the functional relationships among genes may have a negative effect on the accuracy of function predictions. Furthermore, some experiments may be redundant; e.g. replicate the same biological condition or show little differences in terms of the transcriptome profile. Therefore, it is necessary to trim the dataset that is used for construction of the coexpression network in order to remove redundant and uninformative experiments. To this end, we used a heuristic algorithm for selection of the best subset. This algorithm tries to iteratively find experiments whose exclusion can actually improve the accuracy and coverage of the coexpression network. It should be noted that although these ‘excluded’ experiments may have a negative effect on the ‘overall’ accuracy of function predictions, they may provide specific information for particular pathways, as we will show in the results.

### Gene function prediction based on the coexpression network

The functions of currently uncharacterized genes can be predicted based on their association with genes of known functions in the coexpression network. Briefly, if a particular gene is coexpressed with several genes that have a shared function, that gene is also most likely involved in the same function. We calculated a p-value for each gene-pathway pair, so that a small p-value would reflect a significant association between the gene and the pathway. Uncharacterized genes were assigned to biological pathways if their association had a p-value that corresponded to at least 80% precision, meaning that at least an estimated 80% of the predictions are correct.

### Identification of conserved coexpression linkages among genes

Genes with related functions have usually conserved their coexpression through evolution. Thus, if two genes are coexpressed in more than one organism, there is a higher chance that these genes are functionally related [3]. We identified 5300 orthologs of *T. brucei* genes in the closely related organism *Leishmania infantum* based on reciprocal best BLASTP hits with e-values <1×10^−6^. The coexpression value in *L. infantum* was calculated for gene pairs based on a collection of previously reported data from three different studies [15]–[17]. Each pair of conserved genes could then be assigned two values: their Pearson correlation coefficient based on *T. brucei* microarray data, and their Pearson correlation coefficient based on *L. infantum* microarray data. Two genes have a conserved coexpression relationship if both of these values are greater than specified cutoffs (different cutoffs can be used for each organism). The cutoffs were chosen so that the conserved coexpression network would have maximum coverage of *T. brucei* proteins with a precision of at least 50%.

### Identification of potential regulatory motifs in UTRs

We used a previously reported regulatory element discovery method, FIRE, which has been shown to have a close-to-zero false discovery rate and provides a wealth of information about each of the discovered motifs [18]. *T. brucei* genes were clustered based on the data of the three microarray studies [19], and the gene clusters along with either complete or truncated 3′ UTR sequences were submitted to FIRE with default parameters. We only discuss the results of running FIRE on truncated sequences in this paper; the complete set of results can be found at http://webpages.mcgill.ca/staff/Group2/rsalav/web/Suppl/20100109/index.htm.

## Results and Discussion

### A coexpression network of *T. brucei* genes

**Figure 1:**
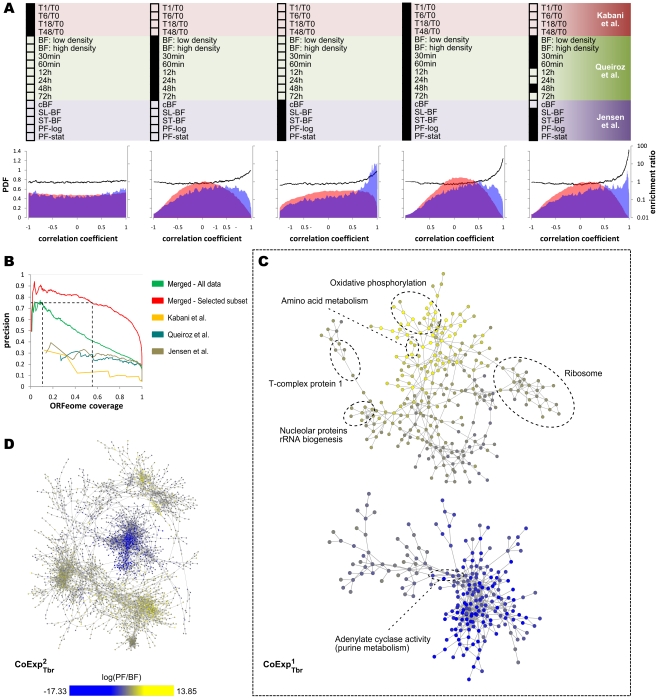
Integration of microarray data for identification of functional linkages among genes. (**A**) The correlation coefficients between genes were calculated for each *T. brucei* dataset separately, for the combination of the three datasets, and for a selected subset of the experiments. The probability density function (PDF) of correlation coefficients among functionally associated and non-associated genes is shown by blue and red, respectively. It can be seen that the data from the work by Kabani et al. [9] are poorly correlated with functional linkages. This is while the other two datasets from Queiroz et al. and Jensen et al. [10], [11] can discriminate functionally linked gene pairs based on the higher correlations of their expression profiles. Consequently, the procedure that we used for selection of the best subset of the experiments automatically excluded the data from Kabani et al. [9], while retaining most of the experiments from the other two datasets (the right panel). The enrichment of functional linkages at a given correlation coefficient, shown by the thick black line, was calculated by dividing the values of the two PDFs. (**B**) Precision (positive predictive value, PPV) vs. ORFeome coverage for prediction of functional linkages based on coexpression is shown in this graph. ORFeome coverage is defined as the fraction of ORFs (open reading frames) with associated expression profiles that are coexpressed with at least one other ORF. By decreasing the threshold for identification of coexpressed pairs, more ORFs are included in the network, but the fraction of coexpression relationships that reflect functional linkages (i.e. precision) decreases. At a precision of 0.75, CoExp^1^_Tbr_ and CoExp^2^_Tbr_ include 10.7% and 55.4% of *T. brucei* ORFeome, respectively. The correlation coefficient cutoff for CoExp^1^_Tbr_ is 0.94 and for CoExp^2^_Tbr_ is 0.957. (**C**) In CoExp^1^_Tbr_, functionally related genes cluster together. A global view of CoExp^2^_Tbr_ is also provided in panel (**D**). Stage-specific expressions are shown by node colors, with yellow for PF-specific and blue for BF-specific proteins. These two networks are provided in Supplementary Dataset S1 and can also be downloaded at http://webpages.mcgill.ca/staff/Group2/rsalav/web/Suppl/20100109/index.htm.

We calculated the pairwise correlation coefficients of mRNA expression profiles for ∼2.8×10^7^ gene pairs in each of the three *T. brucei* microarray datasets as well as in a combined dataset. As Figure 1A shows, if each dataset is considered separately, only a minor enrichment of functionally associated genes can be observed at high correlation coefficients. Nonetheless, when the three datasets are merged, the enrichment ratio of functional linkage between coexpressed genes increases drastically, reaching as high as ∼20 for gene pairs with correlation coefficients >0.90. This can be further improved by objectively selecting the set of experiments that are used for calculating correlation coefficients: removing all the experiments from ref. [9], three out of eight experiments from ref. [10] and one of the five experiments from ref. [11] could increase the enrichment of functionally associated genes up to three-fold for gene pairs with correlation coefficients >0.95 (Figure 1A). This also significantly improved the accuracy of predicting functionally associated gene pairs (Figure 1B). However, as we will show later, while the trimmed dataset is generally more successful in identification of functionally associated genes, the non-trimmed dataset can better identify genes of particular functions, such as oxidative phosphorylation.

Using the combined microarray datasets, we constructed two coexpression networks of *T. brucei* each with an estimated precision of 75% (Figures 1C and 1D and Supplementary Dataset S1). We call the network that is obtained from all microarray experiments CoExp^1^_Tbr_ and the network that is obtained from the selected subset of experiments CoExp^2^_Tbr_. These networks encompass 1280 and 10247 connections among 799 and 4148 *T. brucei* genes, respectively. Most of these genes have no known function (49% in CoExp^1^_Tbr_ and 59% in CoExp^2^_Tbr_ are annotated as hypothetical proteins).

The CoExp^1^_Tbr_ network consists of two main clusters, one with a large number of bloodstream form (BF)-specific genes and one with mostly procyclic form (PF)-specific genes. Some protein complexes and functional modules can be readily distinguished in the sub-network that has most of the PF-specific proteins, as shown in Figure 1C. This modularity of the network should allow us to predict the functions of currently uncharacterized genes. For example, Tb927.10.4880 (formerly identified as Tb10.70.2320), which is currently annotated as “hypothetical conserved”, is located within a complex that corresponds to cytochrome c oxidase. This is congruent with the recent reports showing that this protein co-purifies with cytochrome c oxidase complex [20].

Based on visual inspection, the sub-network with BF-specific proteins has notably less modularity compared to the PF-enriched sub-network. Although several functions are enriched among BF-specific genes (Supplementary Figure S1), they are not represented adequately in this coexpression network due to its low coverage. However, as expected from the higher coverage of CoExp^2^_Tbr_, this network contains more BF-specific genes. It can be anticipated that upon the availability of more microarray data, both the coverage and the precision of the coexpression network will be even further improved and, consequently, a more modular and thorough coexpression network will emerge. Nonetheless, the current networks can be used to predict the functions of many currently hypothetical *T. brucei* genes, as explained in the next section.

### Pathways can be predicted based on coexpression networks of *T. brucei*

**Figure 2:**
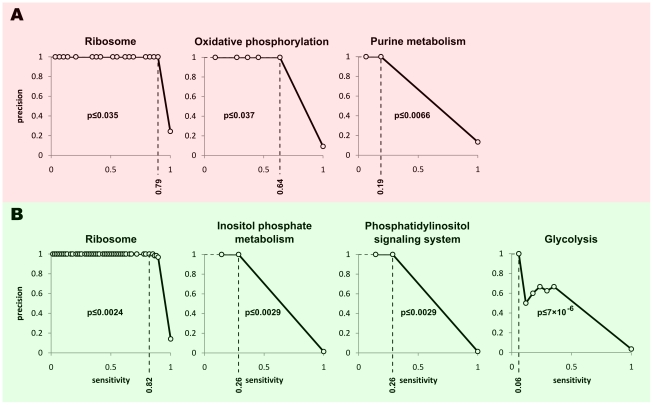
Function prediction based on *T. brucei* coexpression networks. Precision-recall curve for each function is plotted separately. Recall or sensitivity for a particular pathway is defined as the fraction of genes of that pathway within the coexpression network whose function is correctly predicted. Precision indicates the fraction of the predictions that are correct. The CoExp^1^_Tbr_ network can successfully predict ribosome, oxidative phosphorylation, and purine metabolism genes (**A**), while CoExp^2^_Tbr_ is best at predicting ribosome, inositol phosphate metabolism, phosphatidylinositol signalling system, and glycolysis genes (**B**). The p-value thresholds were chosen to be at most 0.05 and result in a precision of at least 0.8. See Supplementary Tables S1 and S2 for complete set of predictions.

As Figure 1C shows, genes of different functions are clustered together in the coexpression network of *T. brucei*. We used this functional relatedness of coexpressed genes to predict functions of uncharacterized genes within the obtained networks. As shown in Figure 2, each of the CoExp^1^_Tbr_ and CoExp^2^_Tbr_ networks are more successful in finding new genes for different pathways: CoExp^1^_Tbr_ can successfully assign new genes to ribosome, oxidative phosphorylation and purine metabolism, while CoExp^2^_Tbr_ can identify genes that are involved in ribosome, glycolysis, inositol phosphate metabolism and phosphatydilinositol signaling system (the genes involved in the latter two pathways considerably overlap, according to KEGG pathway annotations).

We found that many of the genes whose functions are predicted based on our analysis, although having no annotation in KEGG pathway database, are already annotated in TriTrypDB [13]. These annotations are considerably congruent with our predictions, highlighting the reliability of our approach in predicting gene functions. Examples include several cytochrome c oxidase subunits that are correctly assigned to oxidative phosphorylation and many 40S and 60S ribosomal proteins that are correctly assigned to ribosome. While this provides a proof of concept for the method, it also underpins the limitations of KEGG pathway database as the gold standard for construction of the functional linkage network and subsequent function prediction. For example, the gene Tb927.10.4880, which we mentioned in the previous section, cannot be assigned to any function using KEGG pathway information, since none of its neighbors in the coexpression networks are annotated in KEGG. However, if we manually add the known cytochrome c oxidase subunits of *T. brucei* to the oxidative phosphorylation pathway in the gold standard set, our approach can successfully predict that Tb927.10.4880 is involved in oxidative phosphorylation (p<0.001).

Nonetheless, based on the coexpression networks, we can readily predict the likely pathways and biological processes for many of the currently hypothetical proteins. Some of these predictions are also corroborated with available literature. For example, Tb927.10.9830 (formerly identified as Tb10.6k15.0480), which, based on CoExp^1^_Tbr_, is predicted to be involved in oxidative phosphorylation, has been previously reported to be associated with ATP synthase complex [21]. Tb927.4.4020 and Tb927.10.7090 (formerly known as Tb10.6k15.3640) which are coexpressed with purine metabolism genes have several copies of putative regulatory elements that have been previously reported as purine metabolism-specific 3′ UTR motifs [8]. Also, Tb927.6.2330, which, based on CoExp^2^_Tbr_, is predicted to be associated with ribosome, has an RGG domain which has been shown to interact with several ribosomal proteins [22]. The complete list of our predictions based on CoExp^1^_Tbr_ and CoExp^2^_Tbr_ along with literature information that either support or oppose these predictions can be found in Supplementary Tables S1 and S2. The distribution of these genes in the coexpression networks are shown in Supplementary Figure S2.

We have also used the coexpression networks CoExp^1^_Tbr_ and CoExp^2^_Tbr_ to predict the likely biological processes, molecular functions, and cellular compartments of *T. brucei* genes based on GO annotations of TriTrypDB (Supplementary Tables S3, S4, S5, S6, S7, and S8). The analysis of GO annotations complements the KEGG dataset by expanding the predictions of metabolic pathways and also by providing predictions for other categories. For example, we were able to predict novel genes that are potentially involved in antigenic variation, protein folding, and microtubule-based movement. Also, this analysis showed that many proteins within the same cellular compartments are coexpressed, which is not surprising as cellular compartmentalization loosely reflects functional compartmentalization of proteins. This allowed us to predict the likely localization of many proteins; most notably we were able to find potential membrane proteins, intracellular proteins, and proteins associated with dynein complex (Supplementary Tables S5 and S8).

### Conserved coexpression: A closer look

**Figure 3:**
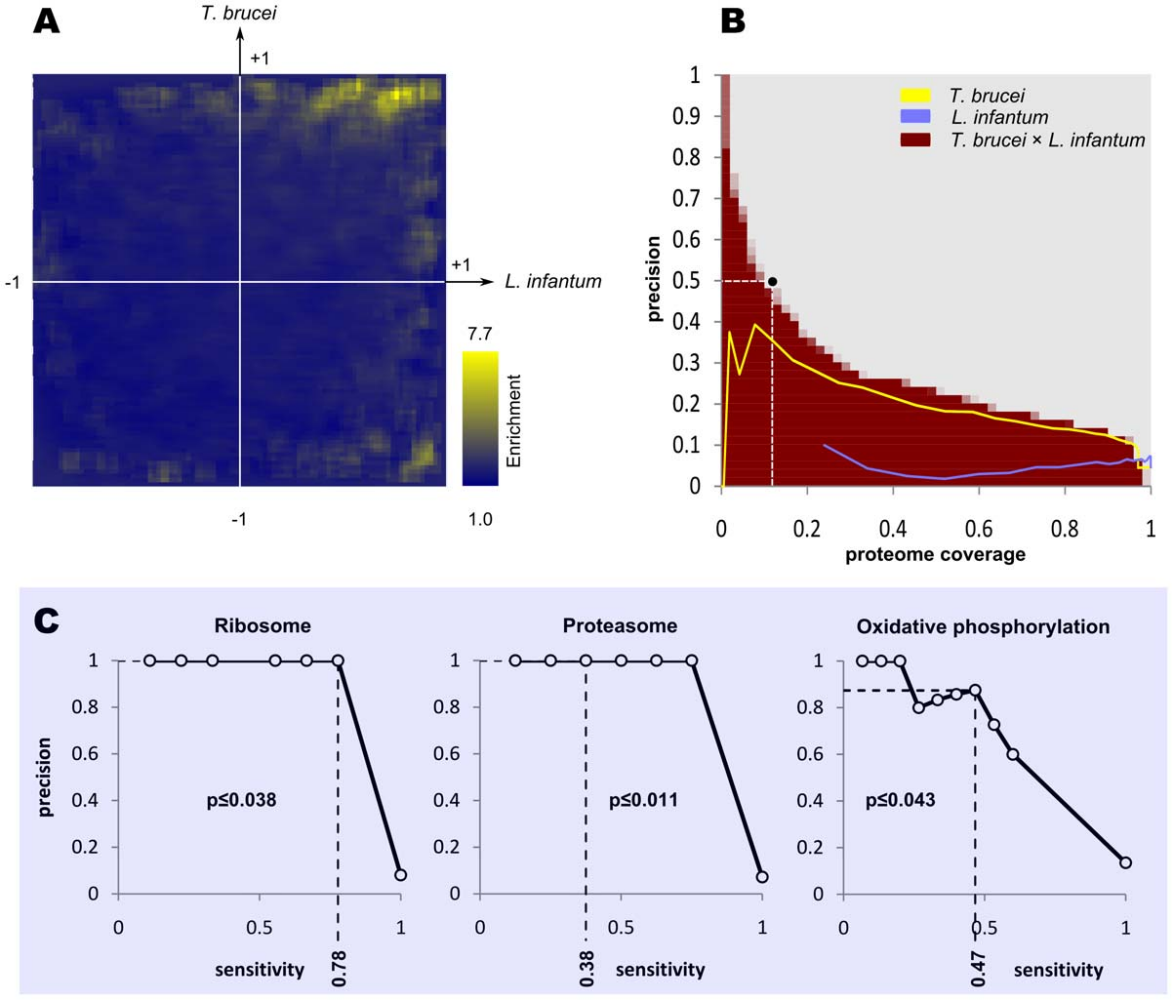
Prediction of functional linkages based on conservation of coexpression. (**A**) Gene pairs that are functionally related are coexpressed in both *T. brucei* and *L. infantum*. Therefore, an enrichment of functional linkages can be observed where correlation coefficients are high for both *T. brucei* and *L. infantum* (the *x*-axis represents the correlation coefficients of gene pairs in *L. infantum*, while the *y*-axis represents that correlation coefficients in *T. brucei*). Enrichment was calculated as Pr(*c_tbr_* = *x*±Δ,*c_lif_* = *y*±Δ|*I*)/Pr(*c_tbr_* = *x*±Δ,*c_lif_* = *y*±Δ|*I′*), where *I* and *I′* represent sets of functionally associated and non-associated gene pairs according to KEGG, respectively. Also, *c_tbr_* and *c_lif_* respectively represent correlation coefficients in *T. brucei* and *L. infantum*. Δ was chosen to be 0.05. (**B**) By considering the conservation of coexpression between *T. brucei* and *L. infantum* (red), we can more accurately predict functional linkages, compared to predictions that are based solely on *T. brucei* data (yellow) or *L. infantum* (light blue). About 50% of gene pairs whose expression profiles have correlation coefficients greater than 0.89 in *T. brucei* and 0.56 in *L. infantum* are estimated to be functionally related (black circle). These gene pairs cover ∼11.9% of all *T. brucei* genes with unambiguous *L. infantum* orthologs. The resultant conserved coexpression network is provided in Supplementary Dataset S1 and can also be downloaded at http://webpages.mcgill.ca/staff/Group2/rsalav/web/Suppl/20100109/index.htm. (**C**) Ribosome, proteasome and oxidative phosphrylation genes can be identified based on the conserved coexpression network. See Supplementary Table S9 for complete set of predictions.

Conservation of coexpression is a much stronger indicative of functional linkages among genes, compared to coexpression in a single organism [3], [23]. Thus, we searched for coexpression associations that were conserved between *T. brucei* and its close relative, *L. infantum*. As Figure 3A shows, in the subset of genes whose orthology between *T. brucei* and *L. infantum* can be unambiguously established, gene pairs that are coexpressed in both *T. brucei* and *L. infantum* are considerably enriched with functional linkages. This property can be used for a more accurate prediction of functional linkages, as shown in Figure 3B: while neither the microarray data of *T. brucei* nor those of *L. infantum* alone can reach a precision higher than 40% for identification of functional linkages among the conserved subset of genes, their combination can yield a wide range of precision and sensitivity values (note that the lower precision of *T. brucei*-only data compared to CoExp^1^_Tbr_ reflects the absence of most of ribosomal proteins from the subset of genes with unambiguous orthologs; see Supplementary Figure S3). We chose our criteria for identification of conserved coexpression relationships so that at least 50% of these relationships reflect functional linkages among genes. This resulted in a conserved coexpression network with 1110 associations among 632 *T. brucei* genes whose orthologs in *L. infantum* could be unambiguously identified (Supplementary Dataset S1). Based on this network, many new genes could be mapped to KEGG pathways (Figure 3C, Supplementary Figure S2). This conserved coexpression network was particularly successful in assigning currently uncharacterized genes to oxidative phosphorylation (Supplementary Table S9). For example, from 17 hypothetical conserved genes that based on this network were predicted to be involved in oxidative phosphorylation, seven genes have been previously identified as potential associated partners or subunits of ATP synthase complex [21]; five others have been reported as mitochondrial proteins, one of which is specifically identified as a mitochondrial membrane protein [24], [25]; and three proteins have a potential regulatory element in their transcript that is also found in the transcripts of many cytochrome c oxidase subunits [26]. This conserved coexpression network could also be used for predicting the likely GO associations of a few *T. brucei* genes (Supplementary Table S10).

These results suggest that functionally related genes are coregulated at mRNA level, most probably through post-transcriptional processes, in different trypanosomatids including both *Trypanosoma* and *Leishmania* genera. Furthermore, this analysis highlights the parallel expression profiling of trypanosomatids as a promising approach that can significantly enhance the functional annotation of all trypanosomatid genomes, including *T. brucei*.

### *Cis*-regulatory element discovery based on clusters of coexpressed genes

**Figure 4:**
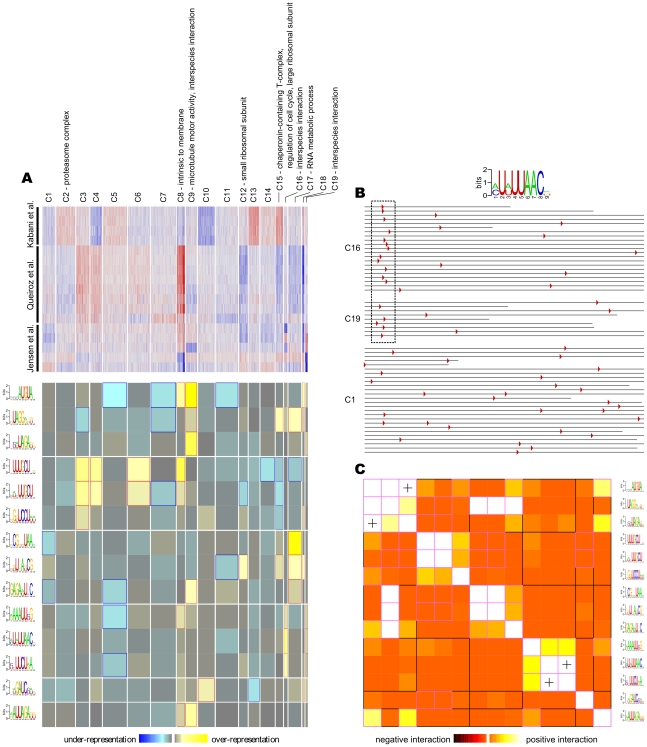
Finding potential regulatory elements based on a combined microarray dataset. (**A**) *T. brucei* genes were grouped into 19 clusters based on their expression profiles (top panel; red: high expression, blue: low expression). FIRE [18] was used to find potential regulatory motifs in the 3′ UTRs (lower panel; yellow and blue represent over-representation and under-representation of a motif within a cluster, respectively). (**B**) The motif [AC]U[AU]UUAAC occurs preferably between nucleotides 40 and 100 downstream of the stop codon in clusters 16 and 19, while its position is random in other clusters, such as cluster 1. Interestingly, both clusters 16 and 19 are enriched with genes involved in interspecies interaction (mostly surface antigens). (**C**) Some motif pairs co-occur in the 3′ UTRs. In this symmetric heat map, each row and each column corresponds to a predicted motif. Light color indicates that the presence of a motif in a 3′ UTR implies the presence of another motif within the same UTR. Significant spatial co-localization between pairs of motifs is shown by “+”. The full set of results along with additional analyses can be found at http://webpages.mcgill.ca/staff/Group2/rsalav/web/Suppl/20100109/index.htm.

Having a collection of microarray datasets, we can study the underlying mechanisms of gene regulation in *T. brucei*. To this end, we first clustered the *T. brucei* genes based on their expression profiles into 19 distinct coexpression groups. The expression patterns within each of these clusters were consistent, and different clusters had unique signatures distinguishing them from each other (Figure 4A). Eight out of the 19 clusters significantly overlapped with at least one Gene Ontology (GO) category, including biological processes, molecular functions, and cellular compartments (p<0.05 with Bonferroni correction for multiple comparisons; all GO terms at all levels were analyzed). We next used FIRE [18] to find potential regulatory motifs in 3′ UTRs across these clusters. FIRE was able to find 14 statistically significant RNA motifs, each over-represented in different gene clusters (Figure 4A). Interestingly, some of these motifs showed a position bias in the clusters in which they were over-represented. For example, the motif [AC]U[AU]UUAAC, which is over-represented among genes that are involved in the interaction of parasite with host, occurs mostly between the 40^th^ and 100^th^ nucleotide after the stop codon of these genes, while showing no position preference in UTRs of genes of other clusters (Figure 4B). Furthermore, many of the found motifs seem to co-occur within the same UTR (Figure 4C). This suggests that they may represent the most conserved parts of a larger, probably structural, RNA motif. This is especially the case for predicted motifs that not only co-occur with each other, but also co-localize at the same part of the 3′ UTR.

### Concluding remarks

The analysis presented in this paper highlights whole-genome transcript profiling as a powerful tool for identification of functional and regulatory modules in *T. brucei*. A comprehensive and high-resolution analysis, however, needs tens to hundreds of different microarray experiments in order to capture the nuances between gene expression patterns of different modules. These experiments should encompass a variety of environmental and genetic conditions, including different stress-inducing culture media and various knockdown/knockout cells. Nonetheless, recent studies suggest that once a large collection of microarray data is available, regulatory and functional modules may be identified even in the absence of such environmental and genetic variations [27]. It should also be noticed that parallel transcript profiling of related organisms may provide more information than excessively thorough transcript profiling of a single organism.

## Supporting Information

Dataset S1 *T. brucei* coexpression networks - This compressed file contains the *T. brucei* coexpression networks and the conserved coexpression network reported in the paper. The enclosed file can be opened using Cytoscape (http://www.cytoscape.org/).
(0.48 MB ZIP)

Figure S1 Functions that are over-expressed in PF or BF *T. brucei*.
(0.51 MB PDF)

Figure S2 Distribution of different KEGG pathways in *T. brucei* coexpression and conserved coexpression networks.
(3.99 MB PDF)

Figure S3 Distribution of conserved proteins in CoExp^1^_Tbr_.
(0.56 MB PDF)

Methods S1 Detailed description of methods.
(0.22 MB PDF)

Table S1 Gene-function associations based on the coexpression network CoExp^1^_Tbr_.
(0.02 MB PDF)

Table S2 Gene-function associations based on the coexpression network CoExp^2^_Tbr_.
(0.01 MB PDF)

Table S3 Prediction of GO biological processes based on the coexpression network CoExp^1^_Tbr_.
(0.05 MB PDF)

Table S4 Prediction of GO molecular functions based on the coexpression network CoExp^1^_Tbr_.
(0.06 MB PDF)

Table S5 Prediction of GO cellular components based on the coexpression network CoExp^1^_Tbr_.
(0.05 MB PDF)

Table S6 Prediction of GO biological processes based on the coexpression network CoExp^2^_Tbr_.
(0.06 MB PDF)

Table S7 Prediction of GO molecular functions based on the coexpression network CoExp^2^_Tbr_.
(0.05 MB PDF)

Table S8 Prediction of GO cellular components based on the coexpression network CoExp^2^_Tbr_.
(0.11 MB PDF)

Table S9 Gene-function associations based on the conserved coexpression network CoExp_Tbr×Lif_.
(0.02 MB PDF)

Table S10 Prediction of GO terms based on the conserved coexpression network CoExp_Tbr×Lif_.
(0.05 MB PDF)
