# Supplementary material for: Functional Genome Annotation by Combined Analysis across Microarray Studies of Trypanosoma brucei
Source: PLoS Negl Trop Dis. 2010 Aug 31;4(8):e810. doi: 10.1371/journal.pntd.0000810 (PMC2930875; doi:10.1371/journal.pntd.0000810)
Supplement: Figure S1 — Functions that are over-expressed in PF or BF T. brucei. (0.51 MB PDF) [file pntd.0000810.s002.pdf]

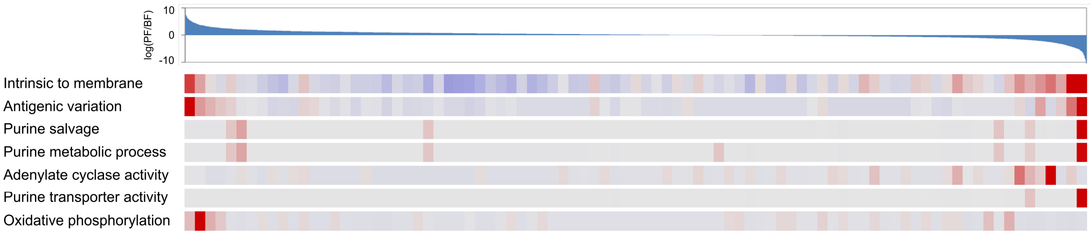

Figure S1. Functions that are over-expressed in PF or BF *T. brucei*. Each row indicates a particular category according to either GO or KEGG, and each column represents a set of genes, whose relative expression in PF and BF cells is indicated in the graph above. Red and blue colors indicate over-representation and under-representation, respectively. Over- and under-representation were calculated based on hypergeometric distribution assumption for the overlap of each functional category with each expression bin. Some categories such as proteins that are intrinsic to membrane or proteins that are involved in antigenic variation are enriched in both PF-specific (left) and BF-specific (right) genes, while metabolism and transport of purines and adenylate cylase activity are mainly over-represented among BF-specific genes, and oxidative phosphorylation is expressed only in PF.
