# Supplementary material for: Functional Genome Annotation by Combined Analysis across Microarray Studies of Trypanosoma brucei
Source: PLoS Negl Trop Dis. 2010 Aug 31;4(8):e810. doi: 10.1371/journal.pntd.0000810 (PMC2930875; doi:10.1371/journal.pntd.0000810)
Supplement: Figure S2 — Distribution of different KEGG pathways in T. brucei coexpression and conserved coexpression networks. (3.99 MB PDF) [file pntd.0000810.s003.pdf]

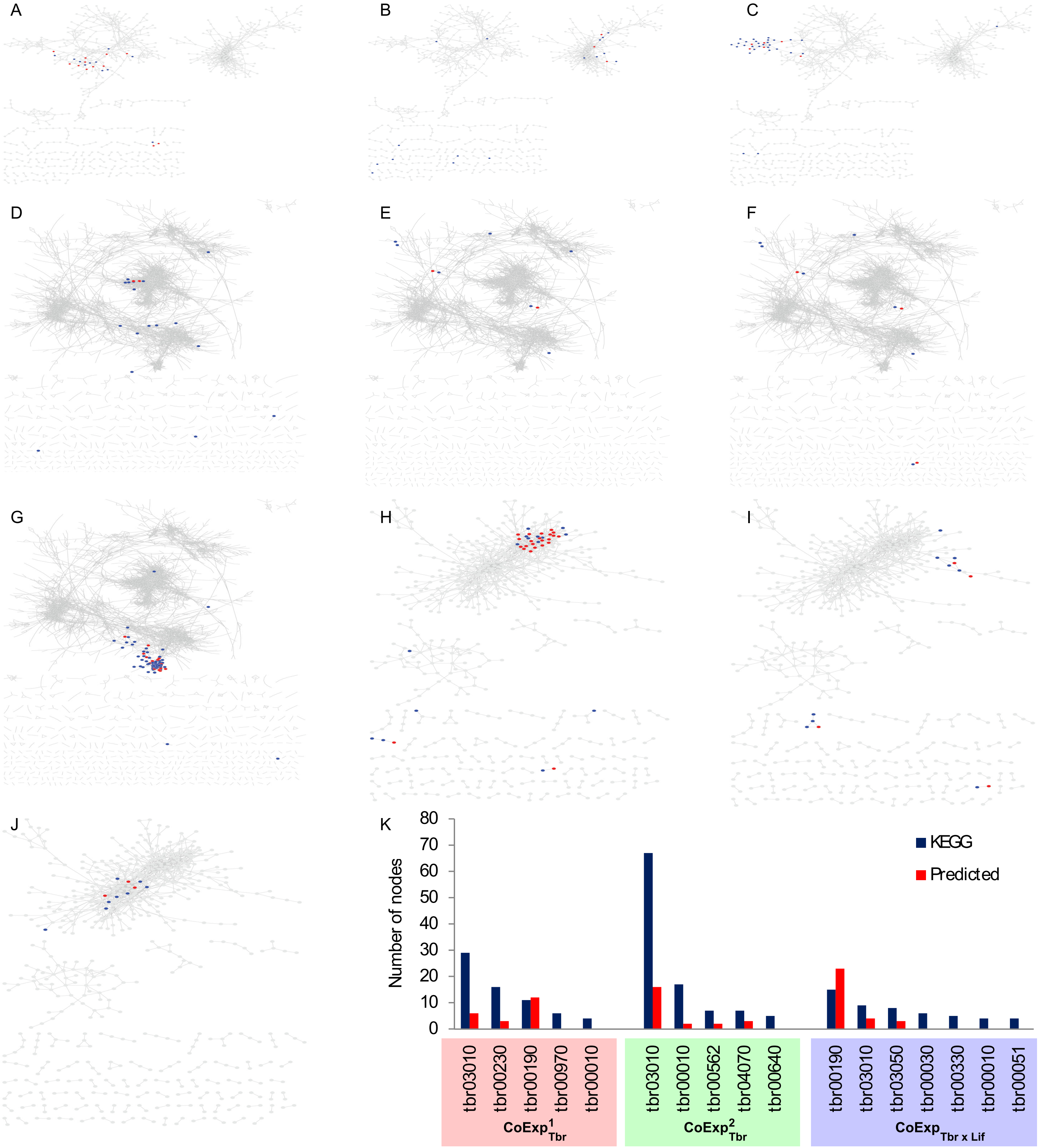

Figure S2. Distribution of different KEGG pathways in *T. brucei* coexpression and conserved coexpression networks. (A) oxidative phosphorylation (tbr00190) proteins in $CoExp^{1}_{Tbr}$; **(B)** purine metabolism (tbr00230) proteins in $CoExp^{1}_{Tbr}$; **(C)** ribosome (tbr03010) proteins in $CoExp^{1}_{Tbr}$; **(D)** glycolysis/gluconeogenesis (tbr00010) proteins in $CoExp^{2}_{Tbr}$; **(E)** inositol phosphate metabolism (tbr00562) proteins in $CoExp^{2}_{Tbr}$; **(F)** phosphatidylinositol signaling system (tbr04070) proteins in $CoExp^{2}_{Tbr}$; **(G)** ribosome (tbr03010) proteins in $CoExp^{2}_{Tbr}$; **(H)** oxidative phosphorylation (tbr00190) proteins in $CoExp_{Tbr×Lif}$; **(I)** ribosome (tbr03010) proteins in $CoExp_{Tbr×Lif}$; **(J)** proteasome (tbr03050) proteins in $CoExp_{Tbr×Lif}$. **(K)** This graph shows the number of proteins that are annotated in KEGG, or whose annotation is predicted, in each network. Only KEGG pathways that could be predicted with at least 80% precision are shown. Note that for some of these pathways no new gene could be predicted.
