# Supplementary material for: Functional Genome Annotation by Combined Analysis across Microarray Studies of Trypanosoma brucei
Source: PLoS Negl Trop Dis. 2010 Aug 31;4(8):e810. doi: 10.1371/journal.pntd.0000810 (PMC2930875; doi:10.1371/journal.pntd.0000810)
Supplement: Figure S3 — Distribution of conserved proteins in CoExp1 Tbr. (0.56 MB PDF) [file pntd.0000810.s004.pdf]

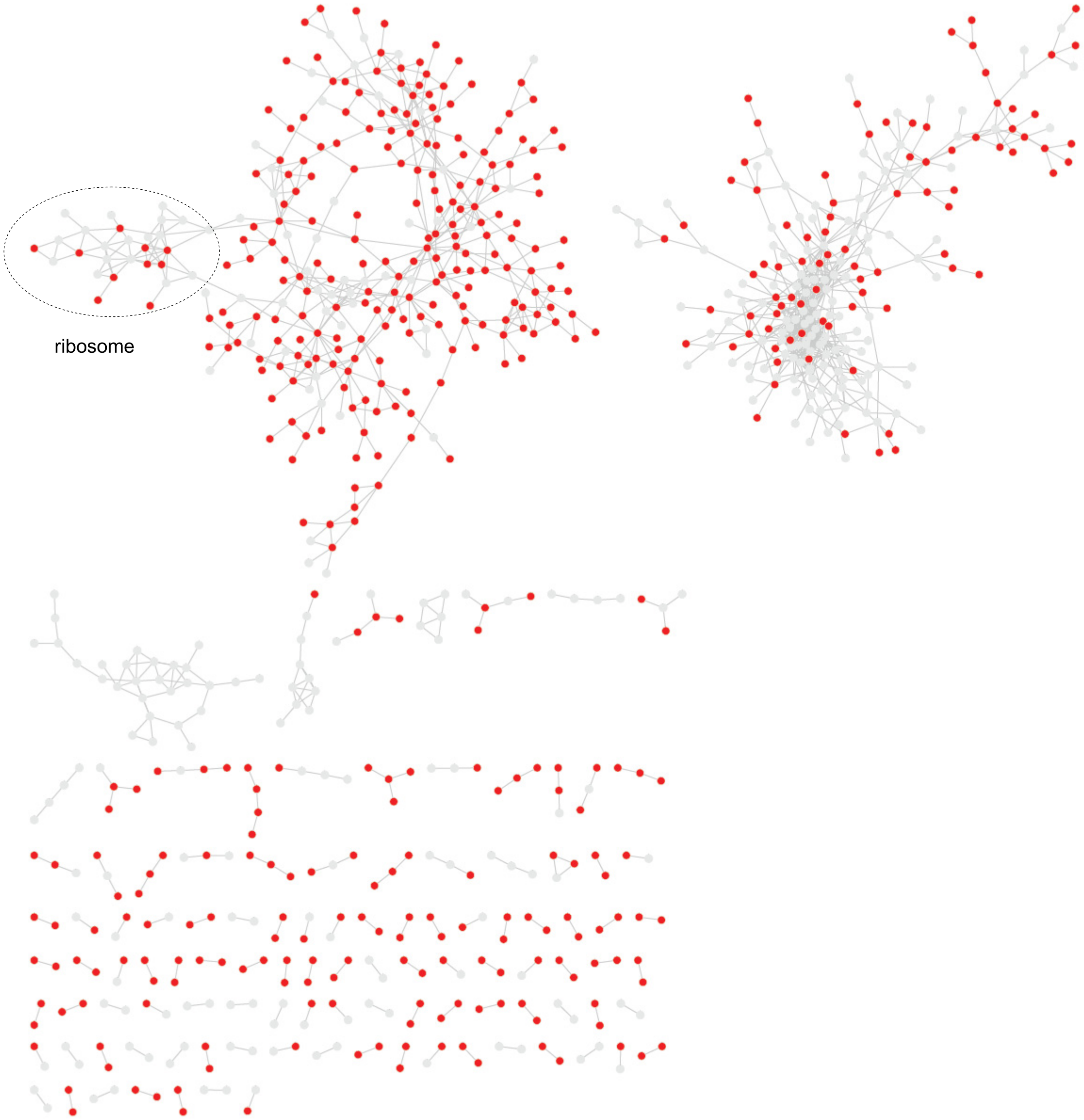

Figure S3. Distribution of conserved proteins in CoExp^{1}$_{\mathbf{Tbr}}$. The orthologs of many *T. brucei* proteins with known KEGG pathways cannot be unambiguously identified in *L. infantum*; this is particularly the case for ribosomal proteins whose sequence similarity to each other prevents unambiguous identification of orthologous partners. Proteins whose *L. infantum* orthologs can be unambiguously identified are shown by red nodes.
