# Supplementary material for: Functional Genome Annotation by Combined Analysis across Microarray Studies of Trypanosoma brucei
Source: PLoS Negl Trop Dis. 2010 Aug 31;4(8):e810. doi: 10.1371/journal.pntd.0000810 (PMC2930875; doi:10.1371/journal.pntd.0000810)
Supplement: Methods S1 — Detailed description of methods. (0.22 MB PDF) [file pntd.0000810.s005.pdf]

Functional genome annotation by combined analysis across microarray studies of *Trypanosoma brucei*

Supplementary Methods

Hamed Shateri Najafabadi[1,2] and Reza Salavati[1,2,3,*]

[1] Institute of Parasitology, McGill University, 21,111 Lakeshore Road, Ste. Anne de Bellevue, Quebec H9X3V9, Canada.

[2] McGill Centre for Bioinformatics, McGill University, Duff Medical Building, 3775 University Street, Montreal, Quebec H3A2B4, Canada.

[3] Department of Biochemistry, McGill University, McIntyre Medical Building, 3655 Promenade Sir William Osler, Montreal, Quebec H3G1Y6, Canada.

* Corresponding author:
Reza Salavati
Institute of Parasitology, Room A-208
21111 Lakeshore Road, Ste. Anne de Bellevue, Quebec H9X 3V9, Canada
Tel: 514-398-7721
Fax: 514-398-7857
Email: reza.salavati@mcgill.ca

Construction of the coexpression networks

Expression values in each microarray experiment, expressed as log ratios of the signal from experimental cDNA to the signal from reference cDNA, were normalized to have a mean of 0.0 and a standard deviation of 1.0. This was done by calculating the average (μ) and standard deviation (σ) for each experiment, and transforming each value by subtracting the average and dividing by the standard deviation: $x=(x'-\mu)/\sigma$, where x' is the original value and x is the transformed (normalized) value. Given two genes α and β from S (S is the set of all genes with associated expression profiles) and their normalized expression values across different experiments of the experiment set E, the coexpression value of α and β can be calculated as the Pearson correlation coefficient of X^E_α and X^E_β, where X^E_α represents the measurements for α in the set E, and X^E_β represents the measurements for β in the set E, as shown in the figure below:

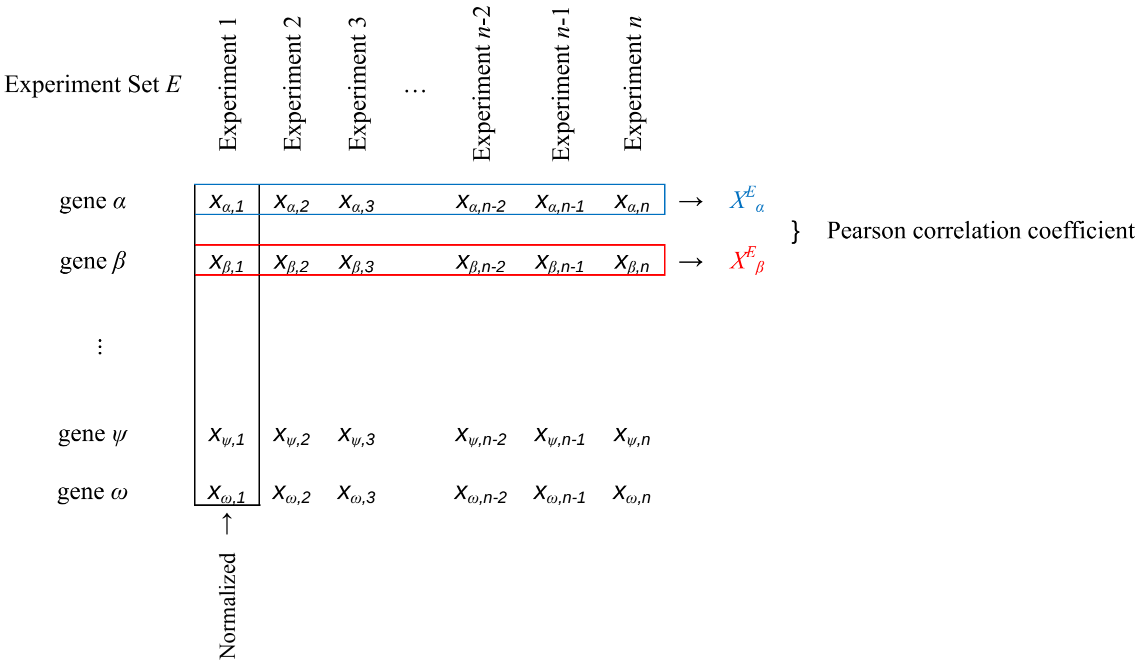

The coexpression network $G^E{}_\theta$ is the set of all gene pairs whose Pearson correlation coefficients, according to the experiment set E, are at least θ:

$G^E{}_\theta=\{(i,j)|\rho(X^E{}_i,X^E{}_j)\geq\theta\}$,

where ρ is the Pearson correlation coefficient function. The set of nodes in the coexpression network $G^E{}_\theta$ is denoted as $N^E{}_\theta$:

$N^E{}_\theta=\{i|\exists j:\rho(X^E{}_i,X^E{}_j)\geq\theta\}$

$|N^E{}_\theta|$ therefore represents the number of nodes in the network $G^E{}_\theta$. The coverage of the network is defined as:

$f^E{}_\theta=|N^E{}_\theta|/|S|$

Thus, $f^E{}_\theta$ indicates what fraction of all genes the network $G^E{}_\theta$ represents. A higher coverage implies that the network can potentially be used for prediction of functions for a larger fraction of *T. brucei* genes with available expression profiles.

The precision of a network in finding functional interactions is calculated by comparing the network to gold standard positive and negative sets. The gold standard positive set I consists of all gene pairs that share at least one function according to KEGG pathway database:

$I=\{(i,j)|F_i\cap F_j\neq\emptyset\}$,

where F_i and F_j represent the set of functions for genes i and j according to KEGG. The gold standard negative set I' includes all gene pairs that do not share any function, given that each gene has at least one annotation in KEGG pathway database:

$I'=\{(i,j)|F_i\cap F_j=\emptyset,F_i\neq\emptyset,F_j\neq\emptyset\}$

The term “tbr01100” (Metabolic pathways) was ignored in all analyses.

The limitations and incompleteness of both I and I' need to be noted: not all *T. brucei* genes with known functions are represented in KEGG; therefore, I is far from complete. Furthermore, the annotations for genes that are present in KEGG may not be complete,

meaning that two genes may actually share a pathway, but this information is missing from KEGG; therefore, I' may contain some gene pairs that should actually belong to I but are mistakenly assumed as negatives.

The positive predictive value (PPV, also referred to as precision) of the network $G^E{}_\theta$ is defined as:

$p^E{}_\theta=|G^E{}_\theta \cap I|/(|G^E{}_\theta \cap I|+|G^E{}_\theta \cap I'|)$

Therefore, $p^E{}_\theta$ estimates the fraction of gene pairs in $G^E{}_\theta$ that are functionally related. We used the area under the curve (AUC) for $p^E{}_\theta(\theta)$ vs. $f^E{}_\theta(\theta)$ as an estimate of how well the experiment set E can reflect the functional linkages among genes. This AUC is here referred to as A^E.

In this study, we used different experiment sets: E_K which is the set of four experiments from ref. [1], E_Q which is the set of eight experiments from ref. [2], E_J which is the set of five experiments from ref. [3], $E_{KQJ}= E_K+E_Q+E_J$, and $\ddot{E}\subseteq E_{KQJ}$. $\ddot{E}$ is chosen so as to result in the maximum A^E:

$A^{\ddot{E}}\geq A^E \ \forall E\subseteq E_{KQJ}$

Since all subsets of E_{KQJ} could not be tested due to computational limitation, we used a heuristic approach to find $\ddot{E}$. A pseudocode for this approach is shown below:

1. Set $\ddot{E}=E_{KQJ}$
2. Create the list $L=\{E'|E'\subseteq E_{KQJ}, |E'|=|\ddot{E}|-1 \vee |E'|=|\ddot{E}|+1\}$
3. Find the E in L that has the maximum A^E
4. If $A^E>A^{\ddot{E}}$ then set $\ddot{E}=E$ and go to step 2
5. Report $\ddot{E}$

Using each of the experiment sets E_{KQJ} and $\ddot{E}$, we defined a coexpression network by selecting the minimum value for cutoff θ that could result in $p_\theta\geq 0.75$ (i.e. precision of at least 75%). The selected value of θ for E_{KQJ} was 0.94 and for $\ddot{E}$ was 0.957. The resulting networks are referred to in the paper as $\text{CoExp}^1{}_{\text{Tbr}}$ and $\text{CoExp}^2{}_{\text{Tbr}}$, respectively.

Identification of conserved coexpression linkages among genes

We identified 5300 orthologs of *T. brucei* genes in the closely related organism *Leishmania infantum* based on reciprocal best BLAST-P hits with e-values $<1\times10^{-6}$. The set of *T. brucei* genes whose *L. infantum* orthologs could be unambiguously identified is referred to as S'. The experiment set E' for *L. infantum* was obtained from three different studies [4,5,6]. The conserved coexpression network $G^{E,E'}_{\theta,\theta'}$ is the set of all gene pairs that are coexpressed according to both experiment sets $E=E_{KQJ}$ (for *T. brucei*) and E' (for *L. infantum*):

$$G^{E,E'}_{\theta,\theta'}=\{(i,j)|i\in S',j\in S',\rho(X^{E}_{i},X^{E}_{j})\geq\theta,\ \rho(X^{E'}_{i'},X^{E'}_{j'})\geq\theta'\},$$

where ρ is the Pearson correlation coefficient function, i' is the ortholog of i in *L. infantum* and j' is the ortholog of j in *L. infantum*. To identify the best θ and θ′ values, we tried all pairs of values so that $\theta\in\{-1,-0.99,-0.98,\ldots,0.98,0.99,1\}$ and $\theta'\in\{-1,-0.99,-0.98,\ldots,0.98,0.99,1\}$. The pair of values that resulted in the maximum coverage of S' and a precision of at least 0.50 was chosen.

To examine the possibility of over-training of θ and θ′ values, we performed a leave-one-out cross-validation, in which each time one gene pair (l,k) was left out, the best θ and θ′ values were determined using the remaining gene pairs, and the left out gene pair was evaluated using these values. If $\rho(X^{E}_{l},X^{E}_{k})\geq\theta$ and $\rho(X^{E'}_{l},X^{E'}_{k})\geq\theta'$, the pair (l,k) was added to the cross-validation network G^{x}:

1. Set $G^{x}=\emptyset$
2. For all $\{(l,k)|l\in S',k\in S',(l,k)\in I\cup I'\}$
3.   If $(l,k)\in I$ then $I=I-\{(l,k)\}$
4.   If $(l,k)\in I'$ then $I'=I'-\{(l,k)\}$
5.   Find the values for θ and θ′ using the new I and I'
6.   If $\rho(X^{E}_{l},X^{E}_{k})\geq\theta$ and $\rho(X^{E'}_{l},X^{E'}_{k})\geq\theta'$ then $G^{x}=G^{x}+\{(l,k)\}$
7.   Restore I and I'
8. Report G^{x}

The G^x was found to have a precision of 0.48 and S' coverage of 0.113 which are very close to the values for the conserved coexpression network that is reported in the paper, implying that the procedure used to find the best values for θ and θ′ did not over-train them.

Network-based prediction of gene function

We evaluated the association of each gene with each KEGG pathway using a hypergeometric-based method: Assume that N is the set of nodes in the network G, $C_\alpha \subset N$ is the set of nodes that are connected to the node α (excluding the node α itself), and $M \subset N$ is the set of nodes that have the particular function f^M according to KEGG, again excluding the node α itself:

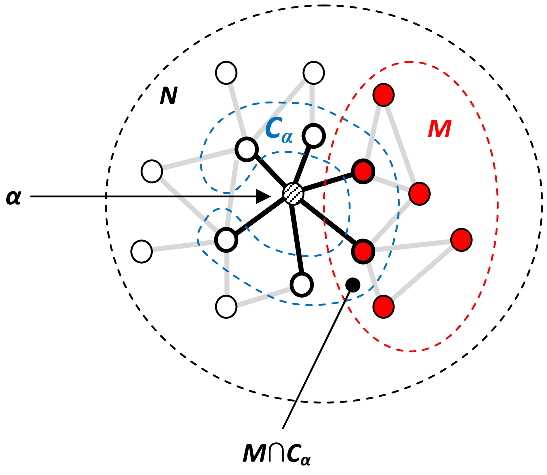

The null hypothesis H_0 is that C_α is independent of M. To evaluate this hypothesis, we assume a hypergeometric distribution for $|M \cap C_\alpha|$:

$\Pr(X \geq x_{obs}|H_0)=\Sigma_x \text{ hypergeo}(x;|N|,|M|,|C_\alpha|)$,

where $x_{obs}=|M \cap C_\alpha| \leq x \leq \min(|M|,|C_\alpha|)$ and “hypergeo” is the hypergeometric distribution function. If H_0 is rejected, the node α is considered associated with M and, thus, with function f^M. Since node α itself is not included in the calculation of the probability value, there is no

need to cross-validate this procedure, as it naturally resembles a leave-one-out cross-validated procedure.

We evaluated the performance of this procedure for each network and each pathway separately. The p-value cutoff for rejecting the null hypothesis was selected to be ≤0.05 and to result in a PPV≥0.80, meaning that at least 80% of predictions are correct.

Identification of potential regulatory motifs in UTRs

T. brucei genes were clustered based on the normalized values of the experiment set E_{KQJ}. We used different clustering approaches: Iclust [7] uses an information-based strategy to cluster the genes into a predefined number of clusters. By default, this number is $\sqrt{|S|}$, where S is the set of all *T. brucei* genes with available expression profiles. Alternatively, we used the standard k-means algorithm with either an initial set of 100 means or an initial set of 30 means. The algorithm converged to 82 and 19 clusters, respectively. Gene clusters along with either complete or truncated 3′ UTR sequences were submitted to FIRE [8] with default parameters. The truncated sequences contained the first 1000bp from the 5′ end of each 3′ UTR. Prior to identification of potential regulatory elements, FIRE removes homologous sequences. In the paper, we only discuss the results of running FIRE on the set of 19 clusters and the truncated sequences; the complete set of results can be found at http://webpages.mcgill.ca/staff/Group2/rsalav/web/Suppl/20100109/index.htm.

References

1. Kabani S, Fenn K, Ross A, Ivens A, Smith TK, et al. (2009) Genome-wide expression profiling of in vivo-derived bloodstream parasite stages and dynamic analysis of mRNA alterations during synchronous differentiation in Trypanosoma brucei. BMC Genomics 10: 427.
2. Queiroz R, Benz C, Fellenberg K, Hoheisel JD, Clayton C (2009) Transcriptome analysis of differentiating trypanosomes reveals the existence of multiple post-transcriptional regulons. BMC Genomics 10: 495.
3. Jensen BC, Sivam D, Kifer CT, Myler PJ, Parsons M (2009) Widespread variation in transcript abundance within and across developmental stages of Trypanosoma brucei. BMC Genomics 10: 482.
4. Ubeda JM, Legare D, Raymond F, Ouameur AA, Boisvert S, et al. (2008) Modulation of gene expression in drug resistant Leishmania is associated with gene amplification, gene deletion and chromosome aneuploidy. Genome Biol 9: R115.
5. Rochette A, Raymond F, Corbeil J, Ouellette M, Papadopoulou B (2009) Whole-genome comparative RNA expression profiling of axenic and intracellular amastigote forms of Leishmania infantum. Mol Biochem Parasitol 165: 32-47.
6. Leprohon P, Legare D, Raymond F, Madore E, Hardiman G, et al. (2009) Gene expression modulation is associated with gene amplification, supernumerary chromosomes and chromosome loss in antimony-resistant Leishmania infantum. Nucleic Acids Res 37: 1387-1399.
7. Slonim N, Atwal GS, Tkacik G, Bialek W (2005) Information-based clustering. Proc Natl Acad Sci U S A 102: 18297-18302.
8. Elemento O, Slonim N, Tavazoie S (2007) A universal framework for regulatory element discovery across all genomes and data types. Mol Cell 28: 337-350.
9. Zikova A, Schnaufer A, Dalley RA, Panigrahi AK, Stuart KD (2009) The F(0)F(1)-ATP synthase complex contains novel subunits and is essential for procyclic Trypanosoma brucei. PLoS Pathog 5: e1000436.
10. Panigrahi AK, Ogata Y, Zikova A, Anupama A, Dalley RA, et al. (2009) A comprehensive analysis of Trypanosoma brucei mitochondrial proteome. Proteomics 9: 434-450.
11. Mao Y, Najafabadi HS, Salavati R (2009) Genome-wide computational identification of functional RNA elements in Trypanosoma brucei. BMC Genomics 10: 355.
12. Czarna M, Jarmuszkiewicz W (2005) Activation of alternative oxidase and uncoupling protein lowers hydrogen peroxide formation in amoeba Acanthamoeba castellanii mitochondria. FEBS Lett 579: 3136-3140.
13. Bouvet P, Diaz JJ, Kindbeiter K, Madjar JJ, Amalric F (1998) Nucleolin interacts with several ribosomal proteins through its RGG domain. J Biol Chem 273: 19025-19029.
14. Lecordier L, Devaux S, Uzureau P, Dierick JF, Walgraffe D, et al. (2007) Characterization of a TFIIH homologue from Trypanosoma brucei. Mol Microbiol 64: 1164-1181.
15. Lee JH, Jung HS, Gunzl A (2009) Transcriptionally active TFIIH of the early-diverged eukaryote Trypanosoma brucei harbors two novel core subunits but not a cyclin-activating kinase complex. Nucleic Acids Res 37: 3811-3820.
16. Lemtiri-Chlieh F, MacRobbie EA, Brearley CA (2000) Inositol hexakisphosphate is a physiological signal regulating the K+-inward rectifying conductance in guard cells. Proc Natl Acad Sci U S A 97: 8687-8692.
17. Bian J, Cui J, McDonald TV (2001) HERG K(+) channel activity is regulated by changes in phosphatidyl inositol 4,5-bisphosphate. Circ Res 89: 1168-1176.
18. Filosa JA, Bonev AD, Straub SV, Meredith AL, Wilkerson MK, et al. (2006) Local potassium signaling couples neuronal activity to vasodilation in the brain. Nat Neurosci 9: 1397-1403.

19. Acestor N, Panigrahi AK, Ogata Y, Anupama A, Stuart KD (2009) Protein composition of Trypanosoma brucei mitochondrial membranes. Proteomics 9: 5497-5508.
20. Mayho M, Fenn K, Craddy P, Crosthwaite S, Matthews K (2006) Post-transcriptional control of nuclear-encoded cytochrome oxidase subunits in Trypanosoma brucei: evidence for genome-wide conservation of life-cycle stage-specific regulatory elements. Nucleic Acids Res 34: 5312-5324.
21. Colasante C, Ellis M, Ruppert T, Voncken F (2006) Comparative proteomics of glycosomes from bloodstream form and procyclic culture form Trypanosoma brucei brucei. Proteomics 6: 3275-3293.
22. Andres JL, Johansen JW, Maller JL (1987) Identification of protein phosphatases 1 and 2B as ribosomal protein S6 phosphatases in vitro and in vivo. J Biol Chem 262: 14389-14393.
