# Supplementary material for: Functional Genome Annotation by Combined Analysis across Microarray Studies of Trypanosoma brucei
Source: PLoS Negl Trop Dis. 2010 Aug 31;4(8):e810. doi: 10.1371/journal.pntd.0000810 (PMC2930875; doi:10.1371/journal.pntd.0000810)
Supplement: Table S1 — Gene-function associations based on the coexpression network CoExp1 Tbr. (0.02 MB PDF) [file pntd.0000810.s006.pdf]

Table S1. Gene-function associations based on the coexpression network $CoExp^1_{Tbr}$. Each gene was evaluated for its association with different KEGG pathways. The significant associations of genes that currently have no annotation in the KEGG pathway database are shown in this table. Old gene ids are brought in parentheses. Note that some of these genes, although not included in the KEGG pathway database, have functional annotations in TriTrypDB, most of which are congruent with the found association. An association with a pathway should be interpreted mainly as a functional linkage, meaning that the corresponding gene is either involved in that pathway, or has a function that is closely related to that pathway and, thus, requires synchronized expression.

	Oxidative phosphorylation	Purine metabolism	Ribosome	TriTrypDB annotation (v2.0)	Notes
Tb927.3.1410	*			Cytochrome oxidase subunit VII	
Tb09.160.1820	*			Cytochrome oxidase subunit V	
Tb927.5.1060	*			Mitochondrial processing peptidase, beta subunit	
Tb927.1.4100	*			Cytochrome oxidase subunit IV	
Tb927.10.9830 (Tb10.6k15.0480)	*			Hypothetical protein, conserved	*a*
Tb927.10.13360 (Tb10.389.0070)	*			Elongation factor Tu	
Tb927.5.2160	*			Hypothetical protein, conserved	
Tb927.4.720	*			Hypothetical protein, conserved	*b*
Tb927.10.11220 (Tb10.26.0790)	*			Procyclic form surface glycoprotein	
Tb927.10.15220 (Tb10.61.1290)	*			Hypothetical protein, conserved	
Tb11.01.1020	*			Hypothetical protein, conserved	
Tb927.10.15960 (Tb10.61.0320)	*			Hypothetical protein, conserved	
Tb927.4.4020		*		Amino acid transporter	*c*
Tb927.5.630		*		Acidic phosphatase	*d*
Tb927.10.7090 (Tb10.6k15.3640)		*		Alternative oxidase	*e*
Tb11.02.2430			***	60S ribosomal protein L17	
Tb11.01.5720			***	Ribosomal protein L18	
Tb927.8.1340			***	60S ribosomal protein l7a	
Tb927.6.5130			*	60S acidic ribosomal protein P2	
Tb11.02.4360			**	40S ribosomal protein S21	
Tb09.211.0340			*	60S ribosomal protein L10	

* $1\times10^{-4} <$ p-value ≤ 0.01
** $1\times10^{-7} <$ p-value $\leq 1\times10^{-4}$
*** $1\times10^{-14} <$ p-value $\leq 1\times10^{-7}$
a Tb10.6k15.0480 has been shown to be associated with ATP synthase complex (PLoS Pathog 2009, 5:e1000436).
b Tb927.4.720 has been reported as a mitochondrial protein (Proteomics 2009, 9:434-450).
c The metabolism of several amino acids, such as Ala, Asp, Glu, His, Gly, Ser and Thr is linked to purine metabolism pathway (KEGG pathway tbr00230). Also, the 3′ UTR of Tb927.4.4020 contains a copy of the motif [ACT]TAA[GT][AG][GT][CT][AC], which has been suggested as a potential purine metabolism-specific regulatory element (BMC Genomics 2009, 10:355).
d Acidic phosphatases are involved in purine nucleoside phosphate catabolism.
e Alternative oxidases are strongly regulated by purine nucleotides (see (FEBS Lett 2005, 579:3136-3140) for an example). In addition, the 3′ UTR of Tb10.6k15.3640 has an instance of the motif [ACGT][AGT][AC]TGCC[AG][GT] and several instances of the motif [ACT]TAA[GT][AG][GT][CT][AC], both of which are suggested as potential regulatory elements specific to purine metabolism (BMC Genomics 2009, 10:355).
