# Supplementary material for: Functional Genome Annotation by Combined Analysis across Microarray Studies of Trypanosoma brucei
Source: PLoS Negl Trop Dis. 2010 Aug 31;4(8):e810. doi: 10.1371/journal.pntd.0000810 (PMC2930875; doi:10.1371/journal.pntd.0000810)
Supplement: Table S2 — Gene-function associations based on the coexpression network CoExp2 Tbr. (0.01 MB PDF) [file pntd.0000810.s007.pdf]

Table S2. Gene-function associations based on the coexpression network $CoExp^{2}_{Tbr}$. Pathways are defined according to KEGG. Refer to Table S1 for more details.

	Ribosome	Inositol phosphate metabolism	Phosphatidylinositol signaling	Glycolysis	TriTrypDB annotation (v2.0)	Notes
Tb927.6.2330	*****				RGG protein	*a*
Tb927.8.1110	*				40S ribosomal protein S9	
Tb927.8.1340	*****				60S ribosomal protein L7a	
Tb927.7.2370	****				40S ribosomal protein S15	
Tb11.01.5720	****				Ribosomal protein L18	
Tb927.6.5130	*				60S acidic ribosomal protein P2	
Tb11.02.4360	*****				40S ribosomal protein S21	
Tb11.02.2430	***				60S ribosomal protein L17	
Tb927.4.3660	***				Hypothetical protein, conserved	
Tb11.01.1465	***				Nascent polypeptide associated complex alpha subunit	
Tb927.5.1110	*				60S ribosomal protein L2, 60S ribosomal protein L8	
Tb927.3.4360	**				40S ribosomal protein S15A	
Tb927.4.5030	*				Protein phosphatase 1	
Tb09.211.0340	****				60S ribosomal protein L10	
Tb11.01.7730	*				Hypothetical protein, conserved	*b*
Tb09.211.3300	*				Peroxin 19 (inferred from mutant phenotype)	*c*
Tb927.7.3530		*	*		Hypothetical protein, conserved	
Tb927.10.16170 (Tb10.61.0090)		*	*		Potassium voltage-gated channel	*d*
Tb11.01.3370			*		Glycosomal membrane protein	*e*
Tb927.7.4500				**	Hypothetical protein, conserved	
Tb927.4.4870				***	Amino acid transporter	

* $1\times10^{-4} <$ p-value ≤ 0.01
** $1\times10^{-7} <$ p-value $\leq 1\times10^{-4}$
*** $1\times10^{-14} <$ p-value $\leq 1\times10^{-7}$
**** $1\times10^{-28} <$ p-value $\leq 1\times10^{-14}$
***** p-value $\leq 1\times10^{-28}$

a RGG domain has been shown previously to interact with ribosomal proteins (J Biol Chem 1998, 273:19025-19029).

b Tb11.01.7730 has been reported to be associated with transcription factor II H (Mol Microbiol 2007, 64:1164-1181; Nucleic Acids Res 2009, 37:3811-3820).

c Tb09.211.3300 has several Pfam domains, such as Pex19 which is essential for peroxisome biogenesis, and LUC7 which is U1 snRNA-associated protein.

d The relationship between Inositol phosphate-mediated signaling and potassium channel activity has been reported in different studies (Proc Natl Acad Sci U S A 2000, 97:8687-8692; Circ Res 2001, 89:1168-1176; Nat Neurosci 2006, 9:1397-1403).

e The 3′ UTR of Tb11.01.3370 contains the motifs [AT]CTTTT[GT]C[ACGT] and [ACG]AGAA[AC]A[AT][AGT]. Both of these motifs have been previously predicted as potential regulatory elements specific to inositol phosphate metabolism and phosphatidylinositol signaling genes (BMC Genomics 2009, 10:355).
