# Supplementary material for: Functional Genome Annotation by Combined Analysis across Microarray Studies of Trypanosoma brucei
Source: PLoS Negl Trop Dis. 2010 Aug 31;4(8):e810. doi: 10.1371/journal.pntd.0000810 (PMC2930875; doi:10.1371/journal.pntd.0000810)
Supplement: Table S3 — Prediction of GO biological processes based on the coexpression network CoExp1 Tbr. (0.05 MB PDF) [file pntd.0000810.s008.pdf]

Table S3. Prediction of GO biological processes based on the coexpression network CoExp$^{1}_{Tbr}$. Although some of these predictions correspond to genes that, according to TriTrypDB v2.0, are already annotated, they have not yet been assigned to any GO biological processes. Each prediction should be interpreted as a functional linkage, meaning that the corresponding gene either belongs to the predicted GO category, or has a function that is closely related to that category and, thus, requires synchronized expression.

	Glycolysis	rRNA processing	Antigenic variation	Protein folding	Cellular protein metabolic process	Regulation of cell cycle	TriTrypDB annotation (v2.0)
Tb927.1.4100	*						Cytochrome oxidase subunit IV
Tb927.10.1560		*					Hypothetical protein
Tb927.4.4790			*				Hypothetical protein
Tb927.3.2520			*				Expression site-associated gene (ESAG) protein
Tb927.3.2500			*				Hypothetical protein
Tb927.3.520			*				Expression site-associated gene (ESAG) protein
Tb927.3.3540				*	**	**	Nucleoporin
Tb927.7.5160					*	*	Deoxyuridine triphosphatase
Tb11.02.0080						*	Hypothetical protein
Tb927.4.1010						*	Hypothetical protein

* $1\times10^{-4} < \text{p-value} \leq 0.01$

** $1\times10^{-7} < \text{p-value} \leq 1\times10^{-4}$
