# Supplementary material for: Functional Genome Annotation by Combined Analysis across Microarray Studies of Trypanosoma brucei
Source: PLoS Negl Trop Dis. 2010 Aug 31;4(8):e810. doi: 10.1371/journal.pntd.0000810 (PMC2930875; doi:10.1371/journal.pntd.0000810)
Supplement: Table S4 — Prediction of GO molecular functions based on the coexpression network CoExp1 Tbr. (0.06 MB PDF) [file pntd.0000810.s009.pdf]

Table S4. Prediction of GO molecular functions based on the coexpression network $CoExp^1_{Tbr}$. Refer to Table S3 for more details.

	Nucleic acid binding	ATP binding	Protein binding	Unfolded protein binding	TriTrypDB annotation (v2.0)
Tb09.211.1040	*				Hypothetical protein
Tb927.10.3910		*			Hypothetical protein
Tb927.7.5160		*			Deoxyuridine triphosphatase
Tb927.3.3540		*	*	*	Nucleoporin

* 1×10^{-4} < p-value ≤ 0.01
