# Supplementary material for: Functional Genome Annotation by Combined Analysis across Microarray Studies of Trypanosoma brucei
Source: PLoS Negl Trop Dis. 2010 Aug 31;4(8):e810. doi: 10.1371/journal.pntd.0000810 (PMC2930875; doi:10.1371/journal.pntd.0000810)
Supplement: Table S5 — Prediction of GO cellular components based on the coexpression network CoExp1 Tbr. (0.05 MB PDF) [file pntd.0000810.s010.pdf]

Table S5. Prediction of GO cellular component based on the coexpression network CoExp$^{1}_{Tbr}$. Refer to Table S3 for more details.

	Cytoplasm	Chaperonin-containing T-complex	Plasma membrane	Cell surface	Integral to membrane	TriTrypDB annotation (v2.0)
Tb09.211.1220	*					Hypothetical protein
Tb927.3.1690	*					Hypothetical protein
Tb927.10.14790	*					Aminopeptidase
Tb927.7.5160		*				Deoxyuridine triphosphatase
Tb11.02.4750			*			Hypothetical protein
Tb09.211.3880			*			Hypothetical protein
Tb927.10.10000				**		Hypothetical protein
Tb927.4.1670				*		Hypothetical protein
Tb09.244.0640					*	Variant surface glycoprotein (VSG
Tb927.3.5690					*	Hypothetical protein
Tb11.01.7530					*	Hypothetical protein
Tb927.4.810					*	Expression site-associated gene (ESAG) protein
Tb927.10.6720					*	Hypothetical protein
Tb927.1.5160					*	Hypothetical protein
Tb927.10.5700					*	Hypothetical protein
Tb927.3.2520					*	Expression site-associated gene (ESAG) protein
Tb927.3.2500					*	Hypothetical protein
Tb927.5.310					*	Hypothetical protein
Tb927.5.1400					*	Hypothetical protein
Tb11.02.1564					*	Leucine-rich repeat protein (LRRP)
Tb927.3.1490					*	Leucine-rich repeat protein (LRRP)
Tb09.211.2060					*	Hypothetical protein
Tb927.8.5080					*	Hypothetical protein
Tb927.3.520					*	Expression site-associated gene (ESAG) protein

* $1\times10^{-4} < \text{p-value} \leq 0.01$

** $1\times10^{-7} < \text{p-value} \leq 1\times10^{-4}$
