# Supplementary material for: Functional Genome Annotation by Combined Analysis across Microarray Studies of Trypanosoma brucei
Source: PLoS Negl Trop Dis. 2010 Aug 31;4(8):e810. doi: 10.1371/journal.pntd.0000810 (PMC2930875; doi:10.1371/journal.pntd.0000810)
Supplement: Table S6 — Prediction of GO biological processes based on the coexpression network CoExp2 Tbr. (0.06 MB PDF) [file pntd.0000810.s011.pdf]

Table S6. Prediction of GO biological processes based on the coexpression network $CoExp^2_{Tbr}$. Refer to Table S3 for more details.

	Translation	Ubiquitin-dependent protein catabolic process	Microtubule-based movement	ATP synthesis coupled proton transport	Antigenic variation	TriTrypDB annotation (v2.0)
Tb927.5.4120	**					Hypothetical protein
Tb09.160.2400	*					Hypothetical protein
Tb927.2.4700	*					Hypothetical protein
Tb927.10.12330	*					Hypothetical protein
Tb11.18.0014	*					Hypothetical protein
Tb927.4.3660	**					Hypothetical protein
Tb927.5.2520	*					Hypothetical protein
Tb927.6.2330	*****					RGG protein
Tb09.211.3300	*					Peroxin 19
Tb11.02.0445	*					Hypothetical protein
Tb927.4.1340	**					Cleavage and polyadenylation specificity factor subunit
Tb11.01.8690	*					Hypothetical protein
Tb927.4.5030	**					Protein phosphatase 1
Tb927.6.2210	*					Hypothetical protein
Tb09.160.3160	*					Hypothetical protein
Tb927.7.640	*					Hypothetical protein
Tb927.8.5990	*					Hypothetical protein
Tb11.01.7730	*					Hypothetical protein
Tb927.7.4120	*					Hypothetical protein
Tb927.3.1370	***					40S ribosomal protein S25
Tb927.10.12310		*				Helicase-like protein
Tb09.211.1270			*			Hypothetical protein
Tb11.02.3880			*			Hypothetical protein
Tb11.02.4120				**		Hypothetical protein
Tb927.3.2180				**		Hypothetical protein
Tb09.142.0310					*	Expression site-associated gene (ESAG) protein
Tb09.211.4820					**	Hypothetical protein
Tb927.10.8980					***	Hypothetical protein
Tb09.160.1440					**	Hypothetical protein
Tb11.02.1470					***	Hypothetical protein
Tb927.4.990					**	Hypothetical protein
Tb11.38.0003					***	Variant surface glycoprotein (VSG)
Tb927.3.5830					***	Expression site-associated gene (ESAG) protein
Tb927.1.5030					**	Leucine-rich repeat protein (LRRP)
Tb09.244.1950					***	Hypothetical protein
Tb927.5.750					**	Hypothetical protein
Tb927.3.2520					**	Expression site-associated gene (ESAG) protein
Tb927.10.5700					*	Hypothetical protein
Tb927.5.390					**	75 kDa invariant surface glycoprotein
Tb11.01.7860					*	Hypothetical protein
Tb927.3.1870					**	Hypothetical protein
Tb927.6.540					***	Gene related to expression site-associated gene 2 (GRESAG2)

* $1\times10^{-4} < \text{p-value} \leq 0.01$
** $1\times10^{-7} < \text{p-value} \leq 1\times10^{-4}$
*** $1\times10^{-14} < \text{p-value} \leq 1\times10^{-7}$
**** $1\times10^{-28} < \text{p-value} \leq 1\times10^{-14}$
***** $\text{p-value} \leq 1\times10^{-28}$
