# Supplementary material for: Functional Genome Annotation by Combined Analysis across Microarray Studies of Trypanosoma brucei
Source: PLoS Negl Trop Dis. 2010 Aug 31;4(8):e810. doi: 10.1371/journal.pntd.0000810 (PMC2930875; doi:10.1371/journal.pntd.0000810)
Supplement: Table S7 — Prediction of GO molecular functions based on the coexpression network CoExp2 Tbr. (0.05 MB PDF) [file pntd.0000810.s012.pdf]

Table S7. Prediction of GO molecular functions based on the coexpression network $CoExp^2_{Tbr}$. Refer to Table S3 for more details.

	Structural constituent of ribosome	Microtubule motor activity	Catalytic activity	TriTrypDB annotation (v2.0)
Tb927.5.4120	**			Hypothetical protein
Tb09.160.2400	*			Hypothetical protein
Tb927.2.4700	*			Hypothetical protein
Tb927.4.3660	**			Hypothetical protein
Tb927.10.3970	*			Hypothetical protein
Tb11.02.0445	*			Hypothetical protein
Tb09.160.3160	*			Hypothetical protein
Tb11.01.7730	*			Hypothetical protein
Tb927.7.4120	*			Hypothetical protein
Tb927.3.1370	***			40S ribosomal protein S25
Tb09.211.1270		*		Hypothetical protein
Tb11.02.3880		*		Hypothetical protein
Tb927.3.3690		*		Flagellar radial spoke protein-like
Tb11.46.0005			**	Hypothetical protein

* $1\times10^{-4} < \text{p-value} \leq 0.01$
** $1\times10^{-7} < \text{p-value} \leq 1\times10^{-4}$
*** $1\times10^{-14} < \text{p-value} \leq 1\times10^{-7}$
