# Supplementary material for: Functional Genome Annotation by Combined Analysis across Microarray Studies of Trypanosoma brucei
Source: PLoS Negl Trop Dis. 2010 Aug 31;4(8):e810. doi: 10.1371/journal.pntd.0000810 (PMC2930875; doi:10.1371/journal.pntd.0000810)
Supplement: Table S8 — Prediction of GO cellular components based on the coexpression network CoExp2 Tbr. (0.11 MB PDF) [file pntd.0000810.s013.pdf]

Table S8. Prediction of GO cellular component based on the coexpression network $CoExp^2_{Tbr}$. Refer to Table S3 for more details.

	Ribosome	Intracellular	Cytoplasm	Chaperonin-containing T-complex	Integral to membrane	Dynein complex	TriTrypDB annotation (v2.0)
Tb11.02.0445	*						Hypothetical protein
Tb927.5.4120	**	**					Hypothetical protein
Tb09.160.2400	*	*					Hypothetical protein
Tb927.2.4700	*	*					Hypothetical protein
Tb927.3.3570	*	*					Hypothetical protein
Tb927.4.3660	**	**					Hypothetical protein
Tb927.6.2330	*****	*****					RGG protein
Tb927.10.3970	*	*					Hypothetical protein
Tb927.6.1130	*	*					Hypothetical protein
Tb09.211.4690	*	*					Hypothetical protein
Tb09.160.3160	*	*					Hypothetical protein
Tb927.8.6250	*	*					Hypothetical protein
Tb11.01.7730	*	*					Hypothetical protein
Tb927.10.160	*	*					Hypothetical protein
Tb11.01.1570	*	*					NUDIX hydrolase
Tb927.7.4120	*	*					Hypothetical protein
Tb927.3.1370	***	***					40S ribosomal protein S25
Tb11.55.0016		*					Hypothetical protein
Tb927.7.6280		*					Hypothetical protein
Tb927.7.3530		*					Hypothetical protein
Tb927.7.3580		*					Protein kinase
Tb09.244.2390		*					Hypothetical protein
Tb11.02.0450		*					Hypothetical protein
Tb927.10.12100		*					RNA-binding protein
Tb927.3.3880		*					Hypothetical protein
Tb927.4.3830		*					Hypothetical protein
Tb927.10.9050		*					Hypothetical protein
Tb09.211.4180		*					Hypothetical protein
Tb927.5.4010		*					Hypothetical protein
Tb11.01.3530		*					Hypothetical protein
Tb927.4.2570		*					Hypothetical protein
Tb927.2.5440		*					Hypothetical protein
Tb09.244.2710		*					Hypothetical protein
Tb09.160.2900		*					PRP3
Tb11.01.6000		*					Hypothetical protein
Tb11.02.4790		*					ATG16/SAP18/CVT11/APG16
Tb09.211.0500		*					Hypothetical protein
Tb927.10.16140		*					Adenylate/guanylate cyclase
Tb927.3.2470		*					Pumilio RNA binding protein
Tb927.7.5150		*					Hypothetical protein
Tb927.10.9180		*					Hypothetical protein
Tb927.1.1400		*					Hypothetical protein
Tb11.01.1910		*					Hypothetical protein
Tb927.7.1450		*					Hypothetical protein
Tb927.6.1180		*					Hypothetical protein
Tb927.4.1980		*					Hypothetical protein
Tb927.1.2200		*					Hypothetical protein
Tb927.8.3090		*					Hypothetical protein
Tb927.5.1770		*					Hypothetical protein
Tb927.8.2000		*					Cyclophilin type peptidyl-prolyl cis-trans isomerase
Tb11.01.6835		*					Hypothetical protein
Tb927.2.2450		*					Ribosomal RNA methyltransferase
Tb927.7.640			**				Hypothetical protein

Tb09.160.2090				*			Hypothetical protein
Tb927.5.2570				*			Translation initiation factor
Tb09.211.1360				*			Hypothetical protein
Tb09.211.0690				**			Hypothetical protein
Tb927.10.14790				*			Aminopeptidase
Tb09.244.2190					*		Hypothetical protein
Tb09.v1.0820					*		Hypothetical protein
Tb927.5.3100					*		Hypothetical protein
Tb927.10.9510					***		Hypothetical protein
Tb09.211.4820					*		Hypothetical protein
Tb09.160.5350					*		Variant surface glycoprotein (VSG)-related
Tb09.142.0320					*		Hypothetical protein
Tb11.02.1564					*		Leucine-rich repeat protein (LRRP)
Tb927.10.1770					**		Hypothetical protein
Tb11.02.3710					*		Hypothetical protein
Tb11.02.1565					*		Hypothetical protein
Tb927.10.6740					*		Hypothetical protein
Tb927.1.5160					***		Hypothetical protein
Tb927.1.5180					*		Hypothetical protein
Tb927.3.580					**		Leucine-rich repeat protein (LRRP)
Tb11.02.1640					*		Kinetoplastid-specific dual specificity phosphatase
Tb11.01.6210					*		Procyclin-associated gene 2-like protein
Tb927.3.2590					*		Hypothetical protein
Tb927.3.570					*		Expression site-associated gene (ESAG) protein
Tb09.244.0640					***		Variant surface glycoprotein (VSG
Tb927.10.8980					**		Hypothetical protein
Tb11.01.6140					*		Hypothetical protein
Tb927.1.1850					*		Hypothetical protein
Tb09.160.1440					*		Hypothetical protein
Tb11.02.1470					**		Hypothetical protein
Tb927.10.1230					**		Hypothetical protein
Tb927.3.3980					*		Hypothetical protein
Tb927.10.14770					*		Protein kinase
Tb927.8.7540					*		Hypothetical protein
Tb927.4.4790					*		Hypothetical protein
Tb927.10.3360					*		Hypothetical protein
Tb11.01.6220					*		Procyclin-associated gene 4 (PAG4) protein
Tb927.10.1040					*		Serine carboxypeptidase III precursor
Tb927.4.990					*		Hypothetical protein
Tb927.7.380					*		Hypothetical protein
Tb11.38.0003					**		Variant surface glycoprotein (VSG)
Tb927.6.1730					*		Hypothetical protein
Tb927.10.8860					*		Hypothetical protein
Tb09.160.4760					*		Hypothetical protein
Tb927.3.5830					***		Expression site-associated gene (ESAG) protein
Tb927.1.5030					*		Leucine-rich repeat protein (LRRP)
Tb927.10.9450					**		Hypothetical protein
Tb927.5.1440					*		Hypothetical protein
Tb927.10.530					*		Hypothetical protein
Tb927.7.190					*		Thimet oligopeptidase A
Tb927.4.1110					*		Hypothetical protein
Tb927.5.3600					*		ATP-dependent DEAD/H RNA helicase
Tb09.244.1950					***		Hypothetical protein
Tb09.160.0360					*		Hypothetical protein
Tb11.01.7380					*		Hypothetical protein
Tb927.3.1490					*		Leucine-rich repeat protein (LRRP)
Tb11.01.7530					*		Hypothetical protein
Tb927.8.980					*		Phosphoacetylglucosamine mutase
Tb927.1.5060					*		Variant surface glycoprotein (VSG)-related
Tb927.5.750					*		Hypothetical protein
Tb927.4.810					*		Expression site-associated gene (ESAG) protein
Tb927.10.1780					*		Hypothetical protein
Tb927.3.2520					**		Expression site-associated gene (ESAG) protein
Tb927.3.5720					*		Hypothetical protein
Tb927.5.4600					**		Expression site-associated gene (ESAG) protein
Tb927.5.1400					***		Hypothetical protein
Tb927.3.560					**		Expression site-associated gene (ESAG) protein
Tb927.10.15440					*		Hypothetical protein
Tb927.3.980					**		Hypothetical protein
Tb927.10.5710					*		Hypothetical protein
Tb927.10.5700					*		Hypothetical protein
Tb927.5.1390					*		64 kDa invariant surface glycoprotein
Tb927.7.6860					*		Expression site-associated gene (ESAG) protein

Tb927.3.2500					*		Hypothetical protein
Tb927.8.4360					*		Hypothetical protein
Tb927.3.5680					*		Variant surface glycoprotein (VSG)-related
Tb11.01.7860					*		Hypothetical protein
Tb927.3.1870					**		Hypothetical protein
Tb927.2.3340					**		Hypothetical protein
Tb927.8.6720					*		Hypothetical protein
Tb927.8.5080					*		Hypothetical protein
Tb927.8.7330					**		Hypothetical protein
Tb927.3.520					**		Expression site-associated gene (ESAG) protein
Tb927.3.5690					***		Hypothetical protein
Tb927.8.7310					*		Hypothetical protein
Tb927.6.540					**		Gene related to expression site-associated gene 2 (GRESAG2)
Tb927.1.2600						*	Pumilio/PUF RNA binding protein 9
Tb09.160.0720						*	Hypothetical protein
Tb11.01.5260						*	Radial spoke protein RSP11
Tb927.1.2760						*	Hypothetical protein
Tb09.244.2050						*	Hypothetical protein
Tb09.244.1650						*	Hypothetical protein
Tb11.01.6390						*	Hypothetical protein
Tb927.3.4510						*	Hypothetical protein
Tb927.10.8780						*	Hypothetical protein
Tb927.1.4310						*	Hypothetical protein
Tb11.02.3880						**	Hypothetical protein
Tb927.3.3300						*	Hypothetical protein
Tb11.02.4640						*	Tubulin-tyrsoine ligase-like protein
Tb927.3.3110						*	Hypothetical protein

* 1×10^{-4} < p-value ≤ 0.01
** 1×10^{-7} < p-value ≤ 1×10^{-4}
*** 1×10^{-14} < p-value ≤ 1×10^{-7}
**** 1×10^{-28} < p-value ≤ 1×10^{-14}
***** p-value ≤ 1×10^{-28}
