# Supplementary material for: Functional Genome Annotation by Combined Analysis across Microarray Studies of Trypanosoma brucei
Source: PLoS Negl Trop Dis. 2010 Aug 31;4(8):e810. doi: 10.1371/journal.pntd.0000810 (PMC2930875; doi:10.1371/journal.pntd.0000810)
Supplement: Table S9 — Gene-function associations based on the conserved coexpression network CoExpTbr×Lif. (0.02 MB PDF) [file pntd.0000810.s014.pdf]

Table S9. Gene-function associations based on the conserved coexpression network CoExp$_{\mathbf{Tbr\times Lif}}$. Pathways are defined according to KEGG. Refer to Table S1 for more details.

	Proteasome	Oxidative phosphorylation	Ribosome	TriTrypDB annotation (v2.0)	Notes
Tb927.10.6800 (Tb10.6k15.3970)	*			Developmentally regulated GTP-binding protein	
Tb927.5.1520	*			Heat shock protein HslVU, ATPase subunit HslU	
Tb927.7.4870	**			Hypothetical protein, conserved	
Tb09.160.0740		*		Hypothetical protein, conserved	
Tb927.10.9830 (Tb10.6k15.0480)		*		Hypothetical protein, conserved	*a*
Tb927.10.8320 (Tb10.6k15.2180)		*		Cytochrome oxidase subunit IX	
Tb11.02.4120		**		Hypothetical protein, conserved	*a*
Tb09.160.1820		*		Cytochrome oxidase subunit V	
Tb927.10.5050 (Tb10.70.2155)		*		Hypothetical protein, conserved	*b*
Tb927.10.4240 (Tb10.70.3010)		**		Hypothetical protein, conserved	*c,d*
Tb927.3.1410		*		Cytochrome oxidase subunit VII	
Tb927.3.700		*		Hypothetical protein, conserved	*d*
Tb927.4.3450		*		Hypothetical protein, conserved	*b,d*
Tb927.8.5120		*		Cytochrome c	
Tb927.10.520 (Tb10.70.7760)		*		Hypothetical protein, conserved	*a*
Tb927.5.1060		**		Mitochondrial processing peptidase, beta subunit	
Tb927.5.2930		**		Hypothetical protein, conserved	*a*
Tb927.4.1360		*		Hypothetical protein, conserved	*e*
Tb11.47.0022		*		Hypothetical protein, conserved	*a*
Tb927.6.590		*		Hypothetical protein, conserved	*b*
Tb927.7.3500		*		Glutathione-S-transferase/glutaredoxin	
Tb927.7.840		*		Hypothetical protein, conserved	*a*
Tb927.5.3090		*		Hypothetical protein, conserved	*a*
Tb927.10.15960 (Tb10.61.0320)		*		Hypothetical protein, conserved	
Tb927.4.720		*		Hypothetical protein, conserved	*b*
Tb927.3.920		*		Hypothetical protein, conserved	
Tb927.5.590			*	Protein phosphatase 1, regulatory subunit	*f*
Tb11.01.5720			*	Ribosomal protein L18	
Tb11.55.0002			*	Protein phosphatase 2C	
Tb927.4.3660			*	Hypothetical protein, conserved	

* $1\times10^{-4} < \text{p-value} \leq 0.01$
** $1\times10^{-7} < \text{p-value} \leq 1\times10^{-4}$
a These proteins have been shown to be associated with ATP synthase complex (PLoS Pathog 2009, 5:e1000436).
b These proteins have been reported to be mitochondrial (Proteomics 2009, 9:434-450).
c These proteins have been reported to be associated with mitochondrial membrane (Proteomics 2009, 9:5497-5508).
d The mRNA of these proteins have the UAG(G)UA(G/U) element, which is also found in the transcripts of many cytochrome c oxidase subunits (Nucleic Acids Res 2006, 34:5312-5324).
e Tb927.4.1360 is suggested as a PF-specific glycosomal protein (Proteomics 2006, 6:3275-3293).
f Protein phosphatase 1 is known to regulate the activity of ribosomal protein S6 (J Biol Chem 1987, 262:14389-14393).
