# Supplementary material for: Functional Genome Annotation by Combined Analysis across Microarray Studies of Trypanosoma brucei
Source: PLoS Negl Trop Dis. 2010 Aug 31;4(8):e810. doi: 10.1371/journal.pntd.0000810 (PMC2930875; doi:10.1371/journal.pntd.0000810)
Supplement: Table S10 — Prediction of GO terms based on the conserved coexpression network CoExpTbr×Lif. (0.05 MB PDF) [file pntd.0000810.s015.pdf]

Table S10. Prediction of GO terms based on the conserved coexpression network CoExp$_{Tbr×Lif}$. Refer to Table S3 for more details.

	Biological Process		Molecular Function	Cellular Compartment		
	Cellular protein metabolic process	Regulation of cell cycle	Catalytic activity	Chaperonin-containing T-complex	Ribosome	TriTrypDB annotation (v2.0)
Tb11.46.0009	**	**		**		Hypothetical protein
Tb927.3.1940			*			Hypothetical protein
Tb927.5.3090			*			Hypothetical protein
Tb927.1.1390			*			Hypothetical protein
Tb927.5.590					*	Protein phosphatase 1

* $1×10^{-4}$ < p-value ≤ 0.01
** $1×10^{-7}$ < p-value ≤ $1×10^{-4}$
